# Healing relationships and the existential philosophy of Martin Buber

**DOI:** 10.1186/1747-5341-4-11

**Published:** 2009-08-13

**Authors:** John G Scott, Rebecca G Scott, William L Miller, Kurt C Stange, Benjamin F Crabtree

**Affiliations:** 1 University of Medicine and Dentistry of New Jersey, Department of Family Medicine, Robert Wood Johnson Medical School, One Worlds Fair Drive, Somerset, NJ 08873, USA; 2 Center for Research in Family Practice and Primary Care, Case Western Reserve University, Department of Family Medicine, 10900 Euclid Avenue, Cleveland, Ohio 44106, USA; 3 Loyola University, Department of Philosophy, 1032 West Sheridan Road 60660, Chicago, Illinois, USA; 4 Lehigh Valley Health Network, Department of Family Medicine, 17^th ^and Chew Street, Allentown, PA 18104, USA; 5 Case Western Reserve University, Departments of Family Medicine, Epidemiology & Biostatistics, and Sociology, 10900 Euclid Avenue, Cleveland, Ohio 44106, USA; 6 Case Comprehensive Cancer Center, 11100 Euclid Avenue #6, Cleveland, Ohio 44106, USA; 7 Cancer Institute of New Jersey, 195 Little Albany Street, New Brunswick, NJ 08901 USA

## Abstract

The dominant unspoken philosophical basis of medical care in the United States is a form of Cartesian reductionism that views the body as a machine and medical professionals as technicians whose job is to repair that machine. The purpose of this paper is to advocate for an alternative philosophy of medicine based on the concept of healing relationships between clinicians and patients. This is accomplished first by exploring the ethical and philosophical work of Pellegrino and Thomasma and then by connecting Martin Buber's philosophical work on the nature of relationships to an empirically derived model of the medical healing relationship. The Healing Relationship Model was developed by the authors through qualitative analysis of interviews of physicians and patients. Clinician-patient healing relationships are a special form of what Buber calls *I-Thou *relationships, characterized by dialog and mutuality, but a mutuality limited by the inherent asymmetry of the clinician-patient relationship. The Healing Relationship Model identifies three processes necessary for such relationships to develop and be sustained: Valuing, Appreciating Power and Abiding. We explore in detail how these processes, as well as other components of the model resonate with Buber's concepts of *I-Thou *and *I-It *relationships. The resulting combined conceptual model illuminates the wholeness underlying the dual roles of clinicians as healers and providers of technical biomedicine. On the basis of our analysis, we argue that health care should be focused on healing, with *I-Thou *relationships at its core.

## Background

In a recent essay about his diagnosis of prostate cancer, New York Times editor Dana Jennings wrote, "Doctors tend to default to mere competent professionalism, forgetting to talk directly to the scared flesh-and-blood man bearing the disease[1]." Such criticism of medicine in the United States is not new, but it is becoming increasingly clear that the medical philosophical position that "considers human beings merely collections of organ systems and deposits of disease entities"[2] is not meeting the needs of patients or health-care professionals.

One unspoken philosophical position underlying modern healthcare is a form of Cartesianism[2](p 99), the idea that bodies are machines and that clinicians are technicians whose job it is to repair those machines. While this medical Cartesian reductionism has had undeniable success in advancing biomedical knowledge, and has led to important interventions to relieve human suffering, it is at the same time the root of many of the problems in our health care system.

Pellegrino and Thomasma propose an alternative philosophy of medicine. They define medicine as "a relation of mutual consent to effect individualized well-being by working in, with, and through the body"[2](p 80). Thus, in their system the center of medicine is relationship, and the purpose of that relationship is healing[2](p 177). This is not to deny the importance of technical competence gained from the reductionist scientific enterprise. As Pellegrino and Thomasma point out, "the act of medical profession is inauthentic and a lie unless it fulfills the expectation of technical competence[2](p 213)." Technical competence, however, is a necessary but not sufficient condition for healing. "Competence must itself be shaped by the end of the medical act - a right and good healing action for a particular patient[2](p 213)."

Pellegrino and Thomasma define a healing action as "a right and good decision for this particular patient," and maintain that healing is simultaneously the goal (telos) of clinical medicine and inherent in the nature of the clinical encounter itself[3]. Pellegrino outlines some professional virtues that are necessary to achieve healing actions,[3] but spends little time on the details of how healing relationships are constructed. The purpose of this paper is to explore in detail the nature of the medical healing relationship using the Healing Relationship Model, a conceptual model the authors developed through qualitative analysis of interviews of physicians and patients, and to connect this model to Martin Buber's philosophical work on the nature of relationships. We make the case that this expanded model of healing relationships, in the context of Pellegrino and Thomasma's philosophy, could serve as a useful theoretical basis against which to measure current efforts to reform the U.S. medical system.

### Healing Relationship Model

In previous research, we used a grounded theory approach to develop a conceptual model of healing relationships between clinicians and patients, the Healing Relationship Model[4]. We sought out six "exemplar" physicians, that is physicians who had a special interest in clinician-patient relationships on the basis of awards, publications, reputation and/or word-of mouth. Each physician was asked to select four or five patients whom he/she thought might have experienced healing. Healing was purposely left undefined so that a definition could emerge from the data. Long semi-structured interviews were conducted with each physician and each patient, focusing on the experience of healing or being a healer, as well as on the relationship between patients and their physicians. The interviews were digitally recorded, transcribed and analyzed by a multidisciplinary team. We used an open coding process to tag data excerpts the group identified as interesting. The analysis team read and reread these excerpts in the context of the larger interview to construct case studies describing the nature of the relationship of the clinician-patient dyad. Insights were discussed, refined, and developed into a coherent case study of each physician and all of his/her patients. Case studies were analyzed across physicians to identify common themes and to develop the Healing Relationship Model.

The definition of healing that emerged from the analysis was the following: Healing means being cured when possible, reducing suffering when cure is not possible and finding meaning beyond the illness experience. We identified three essential processes that are necessary to create and sustain healing relationships: valuing, appreciating power and abiding. **Valuing **refers to the emotional bond that forms between clinician and patient, and is characterized by a non-judgmental stance, finding resonance between the clinician and the patient, and being fully present in the moment with the patient. **Appreciating power **is the recognition that the clinician-patient relationship is inherently asymmetrical, and that the clinician's task is to use that asymmetry for the patient's benefit. **Abiding **refers to the time dimension of the clinician-patient relationship and is characterized by personal continuity, the accumulation of caring actions, and a commitment not to abandon the patient.

These three processes lead to three relational outcomes: trust, hope, and a sense of being known. **Trust **consists of a willingness to be vulnerable, a feeling of being well taken care of, and of knowing that promises will be kept. **Hope **is the belief that some positive future beyond present suffering is possible. **Being Known **is the accumulated sense that the clinician knows the patient as a person.

**Figure 1:**
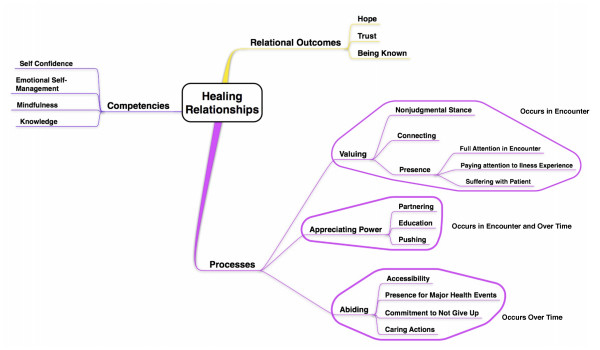
**Healing Relationship Model**.

We also identified clinician competencies necessary for clinicians to participate in healing relationships: self-confidence, emotional self-management, mindfulness, and clinical knowledge. **Self-confidence **is the projection of confidence to the patient of the healer's ability to heal. **Emotional Self-management **is the ability of the clinician to be aware of her own emotional response to the patient's story, and to calibrate that response appropriately. **Mindfulness **in our model is the ability of the clinician to be aware simultaneously and in the moment of the effect of the relationship on both himself and the patient. **Clinical Knowledge **refers not only to the store of knowledge of empirical medicine, but also the ability to synthesize and tailor that knowledge for the benefit of the individual patient. See Figure 1 for a graphic representation of the Healing Relationship Model.

We recognize that there may be many other situations in which healing occurs that are not connected to clinician-patient relationships and that patient contributions to healing relationships are just as important as that of clinicians. In this study we focused on the clinician's role in healing relationships because of its potential to change and improve clinician behavior. We are undertaking another analysis of the data to describe patient characteristics and competencies that promote healing relationships.

### Martin Buber's Philosophy

Martin Buber's work marks the beginnings of a philosophical movement including thinkers like Gabriel Marcel and Emmanuel Levinas that criticizes objectivity as the first or only way of understanding reality. These thinkers emphasize relationality and dialogue over empiricism and objectivity, arguing that objectivity must be understood as a secondary or contrived way of relating to the world. These thinkers critique the modern, Enlightenment understanding of the subject as a separated, substantial, rational entity opposed to a world of 'things in themselves.' The ego, the "I," before it is a separated entity capable of understanding, using, or willing objects, is dependent upon a relation to an un-objectifiable Other.

For Buber, to be is to be in relation, in dialogue. "In the beginning," Buber writes, "is the relation[5](p 18)." This beginning is also a *saying*. To be a human being, for Buber, is to hold oneself in an attitude of relation by saying a "basic word." There are, Buber insists, two basic words, *I-Thou *and *I-It*. One cannot say the word *I *without relating to a world outside the self. These two basic words mark two ways of being in relation to the world. *I-It *relationships are characterized by experiencing and using objects. These are one-way relationships. The *I *of *I-It *relations understands and experiences the world as one composed of objects locatable in space and time. This way of relating to the world makes no distinction between people and things. It is the domain of determinative causality. These relationships are constituted within the horizon of objective temporality, understood as a network of moments passing from future, to present, to past.

*I-Thou *relationships, on the other hand, are two-way relationships based in dialogue. One being encounters another with mutual awareness. *I-Thou *relationships are characterized by what Buber calls presentness. For Buber the present is not "the abstract point between past and future," but "like the eternal now of the mystic, it is the present of intensity and wholeness" and "exists only insofar as meeting and relation exist[6](p 58)." While the *It *of *I-It *relationships is determined by objective temporality, the *Thou *of *I-Thou *relationships resists being ordered in space and time. Buber writes,

"The *Thou *appears in time, but in that of a process that is fulfilled in itself-a process lived through not as a piece that is a part of a constant and organized sequence but in a 'duration' whose purely intensive dimension can be determined only by starting from the *Thou*[5](p 30)."

The *Thou *therefore cannot be understood in terms of its location in a reductive temporal and causal framework.

*I-Thou *relationships live in what Buber calls "the between," the relational space created by the encounter[7](p 241). *I-Thou *relationships are hard to describe, precisely because their nature is not captured in object oriented *I-It *analytic language. The nature of the *I-Thou *relationship is best depicted in Buber's own poetic language:

"If I face a human being as my *Thou*, and say the primary word *I-Thou *to him, he is not a thing among things and does not consist of things. Thus human being is not *He *or *She*, bounded from every other *He *or *She*, a specific point in space and time within the net of the world: nor is he a nature able to be experienced and described, a loose bundle of named qualities. But with no neighbour, and whole in himself, he is *Thou *and fills the heavens. This does not mean that nothing exists except himself. But all else lives in *his *light[5](p 8)."

The experience of *I-Thou *is so powerful that it is not sustainable. In Buber's words,

"It is not possible to live in the bare present. Life would be quite consumed if precautions were not taken to subdue the present speedily and thoroughly[5](p 34)."

Therefore every *I-Thou *relationship must become an *I-It *relationship. In other words, once one experiences the *Thou *as a person with qualities that can be appreciated separately, the evanescent *I-Thou *relationship disappears. Once an *It *has been a *Thou*, however, it always has the potential to become a *Thou *again. Thus, when there has once been an *I-Thou *relationship with another, the ongoing relationship is characterized by a continuous alternation between *I-Thou *and *I-It*[5](p 16-17). Although one cannot create *I-Thou *relationships through force of will, a certain openness to their development must be present on both sides[5](p 11). It is therefore possible for a person to have an *I-It *relationship to another that never becomes *I-Thou*[5](p 34). If a relationship is characterized exclusively by experiencing and using, then the Other never becomes a *Thou*. This is unfortunately the case in those clinician-patient relationships in which diagnosis and treatment are seen as primarily an intellectual endeavor.

Buber is clear that *I-It *relationships are not only inevitable, but also essential to living in the world. It is only through *I-It *relationships that we develop and accumulate knowledge and it is only through them that the scientific ordering of nature can be achieved. The accomplishments of scientific medicine have been realized exclusively through *I-It*. *I-It *relationships, however, only have meaning in the service of *I-Thou*. Buber's words again:

"It is not as though scientific and aesthetic understanding were not necessary; but they are necessary to man that he may do his work with precision and plunge it in the truth of relation, which is above the understanding and gathers it up in itself[5](p 41-42)."

For Buber, overemphasis on *I-It *relationships is dangerous, for increase in our ability to use and experience comes at the expense of our power to enter into relation[5](p 39). It is *I-Thou *relationships which give meaning to our lives and make us fully human[5](p 34). Thus, scientific medicine, disconnected from the relational underpinnings that give it meaning, is a sterile exercise and has potential to do harm. In contrast, *I-Thou *relationships give meaning to medicine and make it a fully human enterprise.

### Pellegrino's Ethical Theory

Pellegrino's neo-Aristotelian ethical theory[3] is rooted in a different ontological framework than that of Buber and Levinas. Pellegrino makes use of Richard Koch's conception of medicine as an Aristotelian *techne*, whose purpose is not to provide knowledge, but to be of use to sick people[8]. While Pellegrino, like Buber, insists on subjugating technical skill to a higher end, Pellegrino's essentialist understanding of the human being differentiates him from many dialogical ethicists. For Pellegrino, the doctor/patient relationship is an encounter between two ontologically definable beings. The relationship arises because one human being lacks some essential component of human flourishing (i.e. health) while another has developed the ability to promote this particular end of human life. Although Pellegrino, like Buber, insists upon the centrality of the doctor-patient relationship for the determination of the end of medicine, this relationship, in Pellegrino's view, is one in which two essentially separate beings encounter one another. For Buber the relation, the between, is primary and constitutive. The I can only be by being in relation. I-Thou and I-It relations are not secondary to a prior ontological reality composed of separately existing entities who encounter one another. While Pellegrino insists that the doctor-patient relationship defines the telos of clinical medicine, his commitment to ontological 'essentialism' and 'realism' distinguish him from Buber who claims that relation precedes and makes possible ontological claims.

### Buber's philosophy and clinician-patient healing relationships Valuing

The three components we have identified as part of valuing (see Figure 1) correspond closely with the characteristics of Buber's description of teacher-student and therapist-client *I-Thou *relationships. The first of these components, "non-judgmental stance", is, for Buber, not simply the uncritical acceptance of the patient as she is, but also the recognition of the potential for positive change. Buber calls this *confirmation*[9](p 38-39).

The other two components of valuing, "connecting" and "presence," are combined in Buber's concept of *inclusion*. This is the ability to experience a relational event from the standpoint of oneself and the other at the same time. Buber carefully distinguishes this concept from empathy. Empathy is an attempt to project oneself into the other person, to experience the event as if one were the other. Inclusion, on the other hand means to simultaneously experience the event as oneself and from the standpoint of the other[7](p 115). It is the job of the educator or psychotherapist (or clinician in our case) to experience at a personal level the effect of the relationship on the patient. Maurice Friedman describes inclusion as follows.

"the psychotherapist, like the educator, must stand again and again not merely at his own pole in the bipolar relation, but also with the strength of present realization at the other pole, and experience the effect of his own action[9](p 32)."

Inclusion, then, necessarily means that the clinician to some degree, experiences the patient's suffering from his own and the patient's standpoint simultaneously. We have called this "suffering with" in the Healing Relationship Model and this is different from the usual conception of empathy.

### Appreciating Power

Clinician-patient relationships are by their nature asymmetrical. In healing relationships, clinicians recognize the inherent asymmetry in the relationship, and use it to benefit the patient. Buber considers such asymmetrical relationships as a special form of *I-Thou *relationship. The examples he uses for this kind of relationship are the teacher-student and therapist-client relationship. In such relationships, full mutuality is neither possible, nor desirable because of the purpose of the relationship. One member of the dyad requires help, and the other professes to be able to help. Thus, by definition, complete mutuality cannot exist in this relationship. Although healing relationships work in both directions, the role of the clinician and the patient in this process are fundamentally different. Friedman, discusses this concept from the standpoint of psychotherapy. We believe it applies equally to any clinician-patient relationship:

"...the difference in position is not only that of personal stance, but of role and function, a difference determined by the very difference of purpose which led each to enter the relationship. If the goal is a common one - the healing of the patient - the relationship to that goal differs radically as between therapist and patient, and the healing that takes place depends as much upon the recognition of that difference as upon the mutuality of meeting and trust. [9](p 31-32)"

Nonetheless, it is incumbent upon the clinician to use this asymmetry to empower the patient to the degree possible permitted by the special nature of the clinician-patient relationship. In the Healing Relationship Model, we identify three ways in which clinicians manage this asymmetry: partnering, educating and pushing.

The first component of appreciating power is "partnering" with the patient in decisions about diagnostic tests and treatment. Maurice Friedman explains Buber's conception of how the built in asymmetry of the therapist-client relationship includes partnering:

"The patient cannot equally well experience the relationship from the side of the therapist or the pupil from the side of the teacher without destroying or fundamentally altering the relationship. This does not mean that the therapist, for example, is reduced to treating his patient as an object, an *It*. The one-sided inclusion of therapy is still an *I-Thou *relationship founded on mutuality, trust, and partnership in a common situation[9](p 31)."

The second component of appreciating power is "educating", by which we mean giving the patient the information he/she needs to both understand and manage his/her illness to the extent that this is possible. For Buber, the act of educating is intimately intertwined with mutuality, trust and partnership. The task of the educator is to select the effective world that the student experiences[7](p 106). It is likewise the task of the clinician to select from the world of medicine that which is relevant to this particular patient and to translate that world in a way that is useful to the patient.

The third component of appreciating power we have termed "pushing." We mean by this that it is sometimes appropriate for the clinician to use her authority to "push" the patient to do something that he may be reluctant to do in the short run, but which will benefit him in the longer term. Buber clearly understood this to be part of confirmation in a therapist-client *I-Thou *relationship. In a dialog with Carl Rogers Buber distinguished between Rogers' concept of acceptance and Buber's concept of confirmation:

"There are cases when I must help him against himself...I can help this man even in his struggle against himself. And this I can only do if I distinguish between accepting and confirming[9](p 30-31)."

The notion of asymmetry in the dialogical relation is one that has had a contentious history in the development of dialogical ethics. Emmanuel Levinas, a contemporary and intellectual descendent of Buber, criticized Buber's portrayal of the dialogical relation as being too rooted in mutuality[10]. For Levinas, the Other is always 'higher' than me. He/She is my master and teacher[11]. According to Levinas, the ethical relation is inherently asymmetrical, but this asymmetry is an inverted version of that described above. The doctor (the 'I' in this instance) is not, in Levinas' view, in a position of power in relation to the patient. Rather, the doctor is the servant of the patient. When I see the patient's face, I am infinitely obligated to this Other who commands me to help. Taking a Levinasian stance, one might, therefore, criticize our embracing of Buber's notion of asymmetry, which is apparently an inversion of Levinas'. The asymmetry of the doctor-patient relationship as we have described it could be seen to limit or even eliminate the role of the patient in constituting the relationship.

In this debate about the asymmetry of the dialogical relation, we must distinguish between two forms of asymmetry. For Levinas, the height of the Other comes from the Other's infinite demand on me. The face of the Other is not constituted by me but constitutes me, invests me with responsibility. The asymmetry of the doctor-patient relationship as we have described it, is an asymmetry of a different sort. The doctor is in a position of 'power' because the patient is scared, vulnerable, or in pain, and the doctor has a certain kind of knowledge and a certain set of skills that may help the patient recover. This latter kind of asymmetry is not, however, incompatible with Levinas' understanding of the asymmetry of the ethical relation. While Levinas describes the Other as my teacher and my master, the Other is also, according to Levinas, the stranger-naked, destitute, and hungry. I must serve the Other because the Other needs me. Thus, the asymmetry that we have described in the doctor-patient relationship is not in direct conflict with a Levinasian understanding of the infinite height of the Other. The Other is both my master and the beneficiary of my wealth.

While it is important to recognize the differences between Levinas and Buber, a complete discussion of the nuances of this debate lies beyond the scope of this paper. What is important for our purposes is the recognition that the asymmetry of the relation as we have described it does not leave the patient powerless. Rather, the asymmetry described is a factual reality of the doctor-patient relationship as it is experienced in practice. Healing is, as we have found, facilitated by an awareness of and proper navigation of this inherent asymmetrical dynamic.

### Abiding

The third process of healing relationships in the Healing Relationship Model we have called "abiding" (See Figure 1). By this we mean both continuity of relationship over time, and a commitment to not abandon the patient. Abiding also includes the accumulation of actions, both large and small, that let the patient know that his or her wellbeing is important to the clinician. On a superficial level, this process seems at odds with Buber's insistence that *I-Thou *relationships are not sustainable for long periods of time. Yet Buber also states that every *It *that has been a *Thou *has the potential to become a *Thou *again. Abiding then, in Buber's system means that relationship endures through constant alternation between *I-Thou *and *I-It*. Buber further addresses this aspect of *I-Thou *relationships with respect to an educator's responsibility to his student. The educator must take on the responsibility to serve as an ongoing connection between the student and the world[7](p 116). This is very similar to the way a clinician must take on the responsibility to serve her patient as a continuing connection to the world of medicine.

Abiding does not mean constant availability, but an underlying commitment to be there in the hour of need. Buber identifies this similar aspect of the teacher-student relationship as "the steady potential presence of the one to the other[7](p 116-117)."

### Mindfulness

We have defined mindfulness as the ability of the clinician to be aware in the moment of the effect of relational events on both clinician and patient. This fits with Buber's term *inclusion*[7](p 115), one of the essential components of an *I-Thou *relationship. Inclusion, as we pointed out earlier, requires two components. The clinician must be aware of the effects of relational events on her as a person, while at the same time using all the clues available to her to experience the effect of the same relational events from the standpoint of the patient. In order to be able to accomplish this, the clinician must be fully present in the moment to the patient. This meshes with Buber's concept of *presentness *in *I-Thou *relationships[6](p 58).

### Emotional self-management

Emotional self-management requires mindfulness as defined above but requires the additional ability to calibrate one's emotional response based on what is best for the patient in the moment. In a sense this is similar to William Osler's idea of *Aequanimitas*, a certain calmness in the presence of patient distress[12]. Osler's famous lecture has been misinterpreted by many as suggesting emotional distance, but we believe that it is a more complex skill. The clinician must project calmness in order for the patient to have hope that healing is possible, yet at the same time the clinician must practice what Buber calls inclusion, that is experiencing the patient's distress from the patient's standpoint. Emotional self-management, then, requires a certain amount of analysis of relational events (an *I-It *relationship) as well as the practice of inclusion (an *I-Thou *relationship). This alternation of *I-Thou *and *I-It *is an essential characteristic of healing relationships.

### Effect of healing relationships on clinicians

Our study used exemplar clinicians who had special interest and expertise in developing healing relationships with patients. We found that these clinicians, in contrast to literature describing the pervasive atmosphere of physician burnout and demoralization in primary care[13], enjoyed their work and derived positive energy from their relationships with patients, even though they had been in practice for many years, some in very challenging practice environments. This finding would not be a surprise to Buber. Clinician-patient healing relationships are, as we have pointed out, a special form of *I-Thou *relationship, and as such provide meaning and sustenance to the clinician as well as the patient[9](p 32). We suspect that clinician burnout occurs when clinician-patient relationships are primarily *I-It*[5](p 46).

### What the Healing Relationship Model adds

Not all the components of our conceptual model are directly addressed in Buber's philosophical system. Our conception of trust as an outcome of healing relationships provides a mechanism for Buber's definition of trust, "a contact of the entire being with the one in whom one trusts[6](p 92)." By analyzing interview data, we were able to identify how trust comes to be in clinician-patient relationships, and the components that make it up. Hope, another outcome in our model, is not addressed by Buber at all, yet hope to be healed is an important reason patients enter the relationship in the first place. We have shown that hope arises partly from the clinician's projection of self-confidence, another construct not addressed by Buber, as well as from the clinician's skill in emotional self-management. Similarly, the sense of being known in our model is addressed by Buber in a general way as part of "inclusion," but we have been able to show how the sense of being known develops over time as a result of valuing and abiding.

## Discussion

We developed the Healing Relationship Model from interview data using a grounded theory approach. We purposely did not use any existing theoretical framework in the analysis. The fact, therefore, that a pre-existing philosophical system fits our model so well is remarkable. The synthesis of our model with Buber's philosophy situated within the context of Pellegrino and Thomasma's philosophy of medical practice creates a theoretical framework that is both data-driven and philosophically coherent. Our conceptual model contributes a detailed view of how a particular kind of relationship is constructed and maintained. Buber's work sets this model in a larger theoretical framework about the nature of human relationships and provides a way to integrate the scientific and relational components of clinician-patient encounters. The philosophy of Pellegrino and Thomasma also situates healing relationships at the center of medical practice and provides the moral and ethical arguments for the superiority of this view over mechanistic Cartesianism.

One could argue that our suggestion that clinicians form *I-Thou *relationships with patients in the context of 15 minute visits is an unreasonable expectation and in fact not necessary for most clinician-patient interactions. Clearly, many patients who come to the clinician for preventive care or acute illness do not feel the need for a healing relationship at that time, yet illness and suffering await all of us sooner or later. The patients in our study, most of whom had chronic illnesses, needed and were able to co-construct healing interactions, interactions that we argue are *I-Thou *relationships, with their clinicians within the usual constraints of modern medical care, including 15 minute visits. The clinicians in our study did not regard forming these relationships as a burden, rather they were what gave meaning, joy and satisfaction to their work.

Medicine in the United States today is almost entirely an *I-It *enterprise. Research funded through the National Institutes of Health, like the organization of the institutes themselves, typically is focused on the study of diseases rather than people and on the development of new technology, with only token support of research exploring the nature of clinician-patient relationships, how such relationships are connected to patient outcomes, and how those relationships might be enhanced. It is not surprising that the current movement toward evidence-based medicine based on this kind of evidence is predominantly *I-It *in nature.

Buber well understood the danger of living only in the world of *I-It*. In fact he defines evil as the mastery of *I-It *within an individual or within a society[5](p 46). The only proper place of *I-It *is in the service of *I-Thou*. Once again, Buber says it best in his own words.

"Man's will to profit and to be powerful have their natural and proper effect so long as they are linked with, and upheld by, his will to enter into relation[5](p 48)."

We do not suggest that *I-It *solutions are not necessary for reforming our broken medical system, but that they are not sufficient. As Buber points out in the quote above, without the establishment of *I-Thou *relationships, *I-It *medical technology is disconnected from its purpose and has increased potential for harm. It is only in the service of *I-Thou *that it can be used appropriately.

Buber's understanding of the nature of human relationships and our detailed conceptual model of healing relationships between clinicians and patients, situated in the moral and ethical context of the work of Pellegrino and Thomasma, puts healing and clinician-patient relationships at the center of medicine. Any reform of the U.S. health care system must recognize the proper role of *I-It *relationships as serving healing and *I-Thou *relationships. Scientific medicine is most powerful and least harmful when used in the service of healing.

We could find only two other papers linking Buber's philosophy to the clinician-patient relationship. Cohn[14] comes to very similar conclusions about the relevance of Buber's philosophy to the practice of medicine and the importance of *I-Thou *in the clinician-patient relationship. Abramovitch and Schwartz[15] emphasize the importance of moving between *I-Thou *and *I-It *in the clinical encounter, although their model of the three stages of medical dialog is somewhat more linear and sequential than our work would suggest. What our work adds is the connection of Buber's ideas to a data-derived conceptual model of healing relationships and to an existing philosophy of medical practice.

## Conclusion

Martin Buber's concepts of *I-Thou *and *I-It *provide a useful theoretical framework for situating an empirically derived detailed conceptual model of healing clinician-patient relationships in the larger context of a theory of human relationships. This combined conceptual model illuminates the wholeness underlying the dual roles of clinicians as healers and providers of technical biomedicine and, in the context of the philosophy of medicine advanced by Pellegrino and Thomasma, provides a coherent philosophical platform against which to measure plans for reform of the U.S. medical system.

## Competing interests

The authors declare that they have no competing interests.

## Authors' contributions

JGS carried out the analysis and wrote the initial draft. RGS added the sections on the philosophical context of Buber's and Pellegrino's work and edited the manuscript. WLM, KCS and BFC provided mentoring and extensive editing of the final manuscript. All authors read and approved the final manuscript

## Authors' informations

JGS is a family physician who spent 21 years in private practice before becoming a qualitative researcher and assistant professor of family medicine at Robert Wood Johnson Medical School. RGS is a PhD candidate in philosophy at Loyola University in Chicago. WLM is a family physician and chair of family medicine at Lehigh Valley Health Network. KCS is a family physician and chair of the research division of the department of family medicine at Case Western Reserve University Medical School. BFC is a medical anthropologist and chair of the research division of the department of family medicine at Robert Wood Johnson University Medical School.
